# Facial Skin Microbiota-Mediated Host Response to Pollution Stress Revealed by Microbiome Networks of Individual

**DOI:** 10.1128/mSystems.00319-21

**Published:** 2021-07-27

**Authors:** Lu Wang, Yi-Ning Xu, Chung-Ching Chu, Zehua Jing, Yabin Chen, Jinsong Zhang, Mingming Pu, Tingyan Mi, Yaping Du, Zongqi Liang, Chandraprabha Doraiswamy, Tao Zeng, Jiarui Wu, Luonan Chen

**Affiliations:** a Shanghai Institute of Biochemistry and Cell Biology, Center for Excellence in Molecular Cell Science, Chinese Academy of Sciences, Shanghai, China; b Unilever R&D Shanghai, Shanghai, China; c School of Life Science and Technology, ShanghaiTech University, Shanghai, China; d Center for Excellence in Animal Evolution and Genetics, Chinese Academy of Sciences, Kunming, China; e Key Laboratory of Systems Health Science of Zhejiang Province, Hangzhou Institute for Advanced Study, University of Chinese Academy of Sciences, Hangzhou, China; f Unilever R&D Bangalore, Bangalore, India; g College of Life Sciences, University of Chinese Academy of Sciences, Beijing, China; h CAS Key Laboratory of Computational Biology, Bio-Med Big Data Center, Shanghai Institute of Nutrition and Health, University of Chinese Academy of Sciences, Chinese Academy of Sciences, Shanghai, China; Mayo Clinic

**Keywords:** skin microbiome, direct association, pollution, single-sample network, microbiome network of individual, microbiome network of population

## Abstract

Urban living has been reported to cause various skin disorders. As an integral part of the skin barrier, the skin microbiome is among the key factors associated with urbanization-related skin alterations. The role of skin microbiome in mediating the effect of urban stressors (e.g., air pollutants) on skin physiology is not well understood. We generated 16S sequencing data and constructed a microbiome network of individual (MNI) to analyze the effect of pollution stressors on the microbiome network and its downstream mediation effect on skin physiology in a personalized manner. In particular, we found that the connectivity and fragility of MNIs significantly mediated the adverse effects of air pollution on skin health, and a smoking lifestyle deepened the negative effects of pollution stress on facial skin microbiota. This is the first study that describes the mediation effect of the microbiome network on the skin’s physiological response toward environmental factors as revealed by our newly developed MNI approach and conditional process analysis.

**IMPORTANCE** The association between the skin microbiome and skin health has been widely reported. However, the role of the skin microbiome in mediating skin physiology remains a challenging and yet priority subject in the field. Through developing a novel MNI method followed by mediation analysis, we characterized the network signature of the skin microbiome at an individual level and revealed the role of the skin microbiome in mediating the skin’s responses toward environmental stressors. Our findings may shed new light on microbiome functions in skin health and lay the foundation for the design of a microbiome-based intervention strategy in the future.

## INTRODUCTION

Urban living is associated with increased exposure to environmental stressors, such as particulate matter, volatile organic compounds, and dust fall. Human skin is the first layer of the body’s natural defense against external stressors in urban living. Increasing numbers of epidemiological and mechanistic studies have indicated the clear link between pollution and skin concerns, such as blemishes (1), dermal aging (2), and skin inflammation (3).

The skin microbiome is an integral part of the skin barrier that can affect skin health by modulating host immune responses and the skin barrier function (4). The dysbiosis of the microbiome has been associated with various diseases. Various studies adapted the connectivity and robustness from network theory (5) to investigate the functional importance of the skin microbiome in health and diseases from a microbiome ecological perspective. There is increasing evidence indicating a link between undesirable skin conditions with altered microbial assembly process and a more fragile microbiome network (6, 7). Specifically, a cooccurrence network of the microbiome was found to be affected by environmental factors (8). In a study of the skin microbiome in China, a microbial cooccurrence network was found to be more fragile in urbanized environments where the higher incidence of skin diseases has been observed (6). We hypothesize that the skin microbiome, being the first layer of the skin barrier (4), could mediate the effect of urban living on skin physiology. Until now, little has been known about the directional relationship between the skin microbiome and skin health under environmental stressors.

Mamet et al. (9) reported a potential causal hypothesis between climate, soil property, and microbial beta diversity by mediation analysis. Mamet et al. (10) further introduced the network concept to construct a microbiome network of the population (MNP) and disclosed the mediation relationships between plant, soil, and soil microbiome by mediation analysis. However, the MNP approach is limited for studying the mean value across a population of samples or subjects and cannot elucidate microbe occurrence patterns or microbiome networks at an individual level (10) or characterize the heterogeneous features of each individual in a population. In gene expression studies, several methodologies based on a single-sample network (SSN) framework have been developed to characterize statistical perturbation of a single sample against a group of control samples and have demonstrated an important regulatory pattern of gene networks in clinical settings (11–14). Thus far, there is no such method for a microbial community, which requires different constraints from gene expression networks due to the compositional nature of microbiome sequencing data (15). Simply adopting a traditional SSN method for microbiome network construction may result in spurious correlations due to this compositional effect.

**FIG 1:**
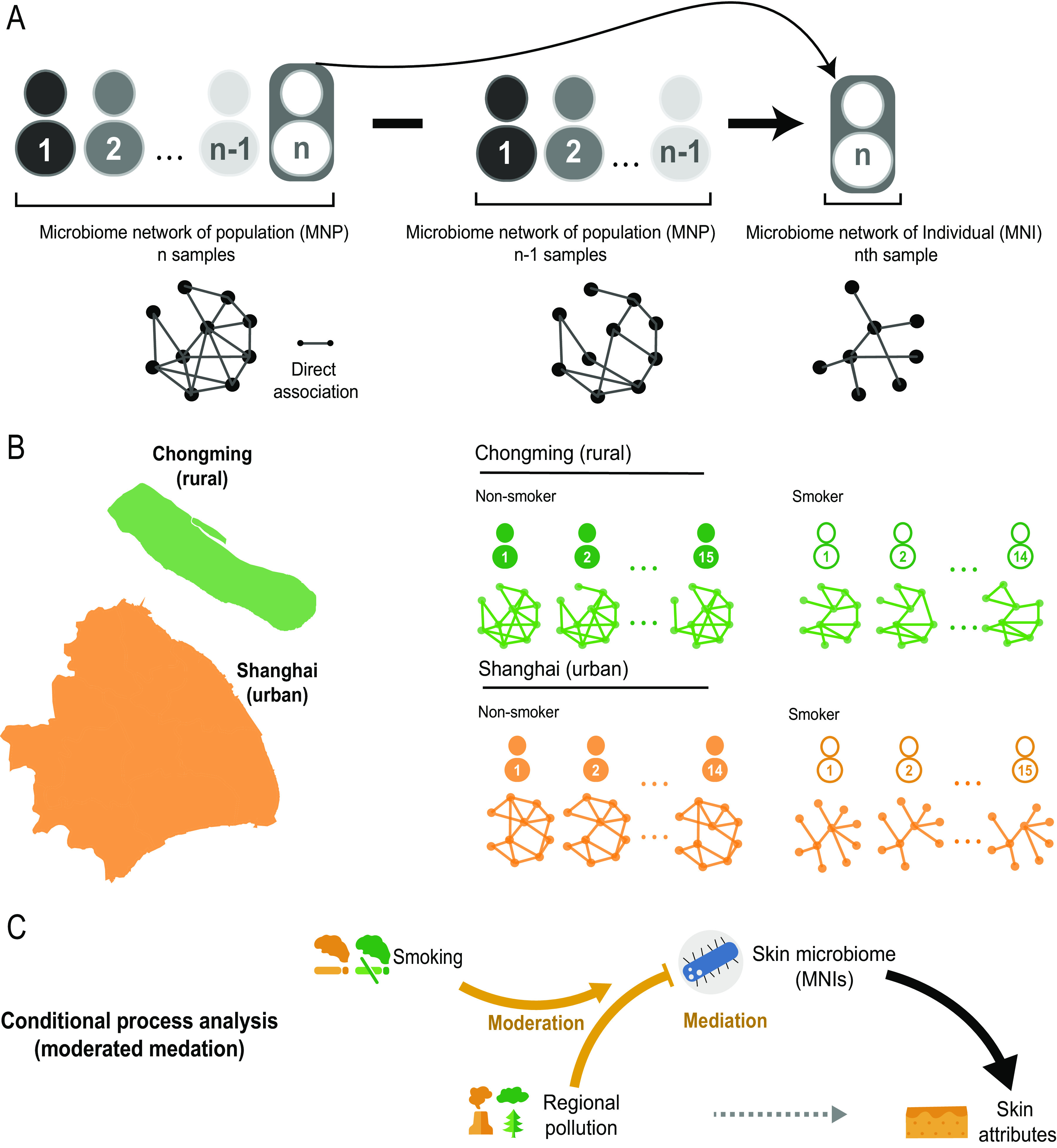
Framework of network analyses by microbiome network of individual (MNI). (A) In order to construct an MNI for each subject/sample, we first built a population network of *n* samples and then eliminated the effect of all but the sample of interest. Next, we repeated this process to obtain an MNI for each sample (detailed information is in Materials and Methods). (B) Samples were collected from 58 subjects, including rural smokers (*n* = 15 from Chongming smokers [CS]), urban smokers (*n* = 14 from Shanghai smokers [SS]), rural nonsmokers (*n* = 14 from Chongming nonsmokers [CN]), and urban nonsmokers (*n* = 15 for Shanghai nonsmokers [SN]). We constructed an MNI for each subject, totaling 58 individual MNIs. (C) The mediation role of the skin microbiome was revealed by MNI analysis for each subject. In particular, the moderated mediation effect of network properties of MNIs was statistically tested using conditional process analysis.

To overcome these methodological limitations and illustrate the dysbiosis of the microbiome under pollution effects on an individual, we developed a new method for constructing a microbiome network of an individual (MNI). Specifically, the MNI method adopted the following principles: a population cooccurrence network (or MNP) generated from a set of samples is a linear combination of cooccurrence networks of individual samples (16), and the interaction of microbes can be represented with partial correlations between microbes based on a precision matrix generated by the SPIEC-EASI framework (17). In other words, an MNI is a network specific for one sample and subtracts the effects of all other samples from MNP (Fig. 1). This is also a direct-association network rather than a traditional Pearson correlation network that mixed both direct and indirect associations between microbes (17). In MNI, we adopted partial correlation, which can eliminate indirect association caused by confounders and inferred only direct association between a pair of nodes/species (17). Intuitively, an MNI is able to provide a snapshot of a microbial community on an individual in terms of the network. Through the construction of MNIs, we could obtain network properties from each subject.

We previously reported that taxi drivers exposed to heavy-traffic-derived pollutants in urban Shanghai city (SH) had weakened skin physical and antioxidant barrier properties compared with those on Chongming island (CM) (18), a rural area of the Shanghai municipality. Here, we further profiled the facial skin microbiome of the two taxi driver cohorts by 16S amplicon sequencing with subgroups of smokers and nonsmokers in both SH and CM (a total of 58 subjects) and constructed an MNI for each subject. Using urban/rural and smoking/nonsmoking as major exposure factors with a conditional process model, we analyzed skin MNPs and MNIs under different pollution conditions. Our analysis revealed that air pollution had an apparent effect on the fragility of the skin microbial network at both the population and individual levels; this fragile microbiome network mediated the skin’s physiological responses to pollution, thus damaging skin health, and a smoking lifestyle further amplified the damaging effects of air pollution on the microbiome and the skin. Our results revealed, for the first time, that environmental stressors exert significant impacts on the network of the microbial community for each subject (Fig. 1), which subsequently influence the skin’s physiological properties.

## RESULTS

Shanghai is a highly urbanized megacity, while Chongming county is a nature reserve outside Shanghai, with a lower population density, lower industrial production, and higher green area coverage. According to the Shanghai Statistical Yearbook (2014, 2015, and 2016), air quality in Chongming is constantly better than that of Shanghai. To study the impact of pollution stress on the facial microbiome, we applied 16S amplicon sequencing to compare facial skin microbiome profiles (upper cheek) of taxi drivers from Shanghai and Chongming, with a 1:1 smoker-to-nonsmoker ratio (all males balanced by age). Among the four groups of Chongming nonsmokers (CN, *n* = 15), Chongming smokers (CS, *n* = 14), Shanghai nonsmokers (SN, *n* = 14), and Shanghai smokers (SS, *n* = 15), the SS group was thought to have the most exposure to pollution stress (air pollution plus cigarette smoke) while the CN group was thought to have the least (Fig. 1). The terminology and notation related to each network are given in detail in Materials and Methods.

### Pollution stress affected the skin microbiome network of the population.

**FIG 2:**
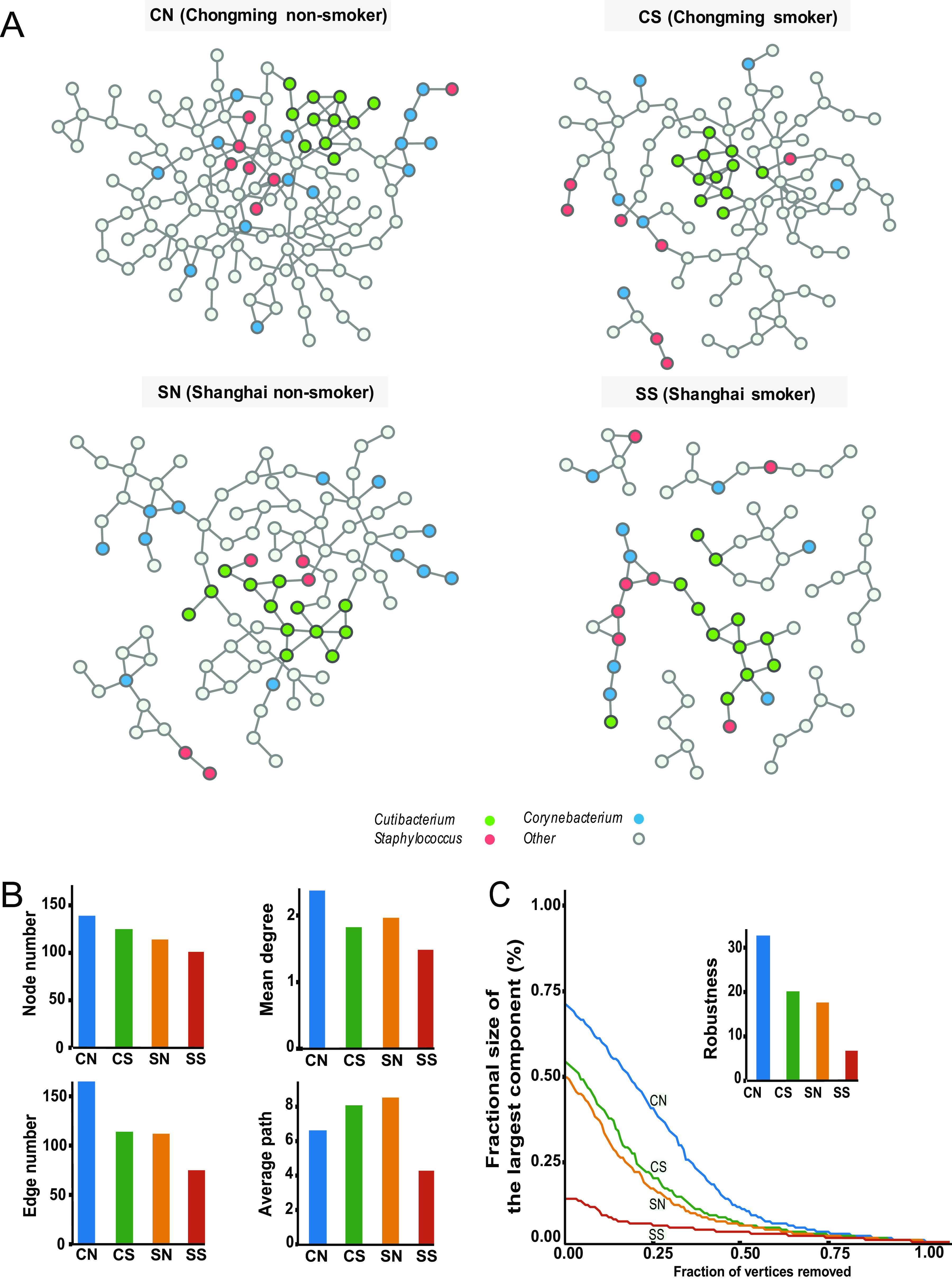
Analysis of the microbiome network of populations (MNP) for each group of subjects under different pollution conditions. (A) MNPs of each group. Each node represents a microbial taxon (OTU). Associations were detected using the SPIEC-EASI method. Clearly, there were significant differences in terms of network between the four groups. Three dominating genera (accounting for over 50% of the total abundance of all species), *Cutibacterium* (green), *Corynebacterium* (blue), and Staphylococcus (red), were colored. (B) Connectivity properties (node number, edge number, mean degree, and average path) of the MNPs of each group. (C) Fragility of the MNP of each group. Fragility was measured by the percentage of remaining nodes in the giant (largest) component, which was indicated on the *y* axis. The percentage of nodes/vertices removed was indicated on the *x* axis. The fragility of each network characterizes the robustness of each network. Both a larger area under the curve and a high robustness value indicate the corresponding network was more robust/less fragile.

Conventional MNP analysis showed that the skin microbiome from a group exposed to less pollution formed a more robust community than that from a group exposed to more pollution (Fig. 2A). We then compared the connectivities and fragilities of MNP across the four groups. The connectivity of the microbiome community was examined based on the MNP attributes of the overall network structure (Fig. 2B). Among the four groups, the MNP of CN had the largest connected component, whereas that of SS had the smallest (Fig. 2A). The node numbers and edge numbers of MNPs were in the order of CN > CS > SN > SS, which indicated higher microbial network connectivity in the lower-pollution groups. An analysis of mean degree (i.e., the mean of degrees for all nodes in one network) agreed with this finding that the CN network had the highest mean degree of any of the groups. The average paths showed an inconsistent trend where SN had the highest value while that from SS had the lowest one (Fig. 2B). The overall trends of MNP attributes were in agreement with previous results (6), indicating that people living in an environment of low urbanization had better connectivity of their microbial networks. In addition, we measured the fragility of these microbial networks by sequentially removing individual nodes (e.g., operational taxonomic units [OTUs]) from the network to simulate system collapse (5). As shown in Fig. 2C, CN had the largest area under the curve, indicating that it had the most robust MNP under this simulated disruption. In contrast, the SS group had the most fragile MNP, suggesting that the skin microbial network becomes more fragile under more severe pollution stress.

We next examined microbial composition under different pollution stresses. Staphylococcus, *Cutibacterium*, and *Corynebacterium* had the highest relative abundances on facial skin from all of these groups (CN, CS, SN, and SS) in Fig. S1A in the supplemental material, which agreed well with reported literature (20). All of the dominant genera (with a mean of relative abundance of >1%) had no significant difference between these four groups (*P* > 0.05). In our study, alpha diversity was assessed by the Shannon index while beta diversity was measured by Bray-Curtis dissimilarity. Both alpha diversity (*P* > 0.05) and beta diversity (false-discovery rate [FDR] > 0.05) showed no significant difference between these four groups (Fig. S1B).

10.1128/mSystems.00319-21.1 FIG S1 Relative abundance and alpha and beta diversity of skin microbiota of CN, CS, SN, and SS. (A) Relative abundance of bacterial genus comprising >1% (mean value of all samples) of the total population. (B) Alpha diversity indicated by Shannon index. (C) Beta diversity indicated by Bray-Curtis dissimilarity. Download FIG S1, PDF file, 0.4 MB. Copyright © 2021 Wang et al. 2021 Wang et al. https://creativecommons.org/licenses/by/4.0/ This content is distributed under the terms of the Creative Commons Attribution 4.0 International license.

Our results indicated that pollution stress did not significantly change the composition or diversity of the microbiome but affected the fragility of the microbial ecosystem, that is, it made the microbiome networks fragile.

### Microbiome network of individual (MNI) revealed the fragility of the skin microbial community under pollution conditions at an individual level.

**FIG 3:**
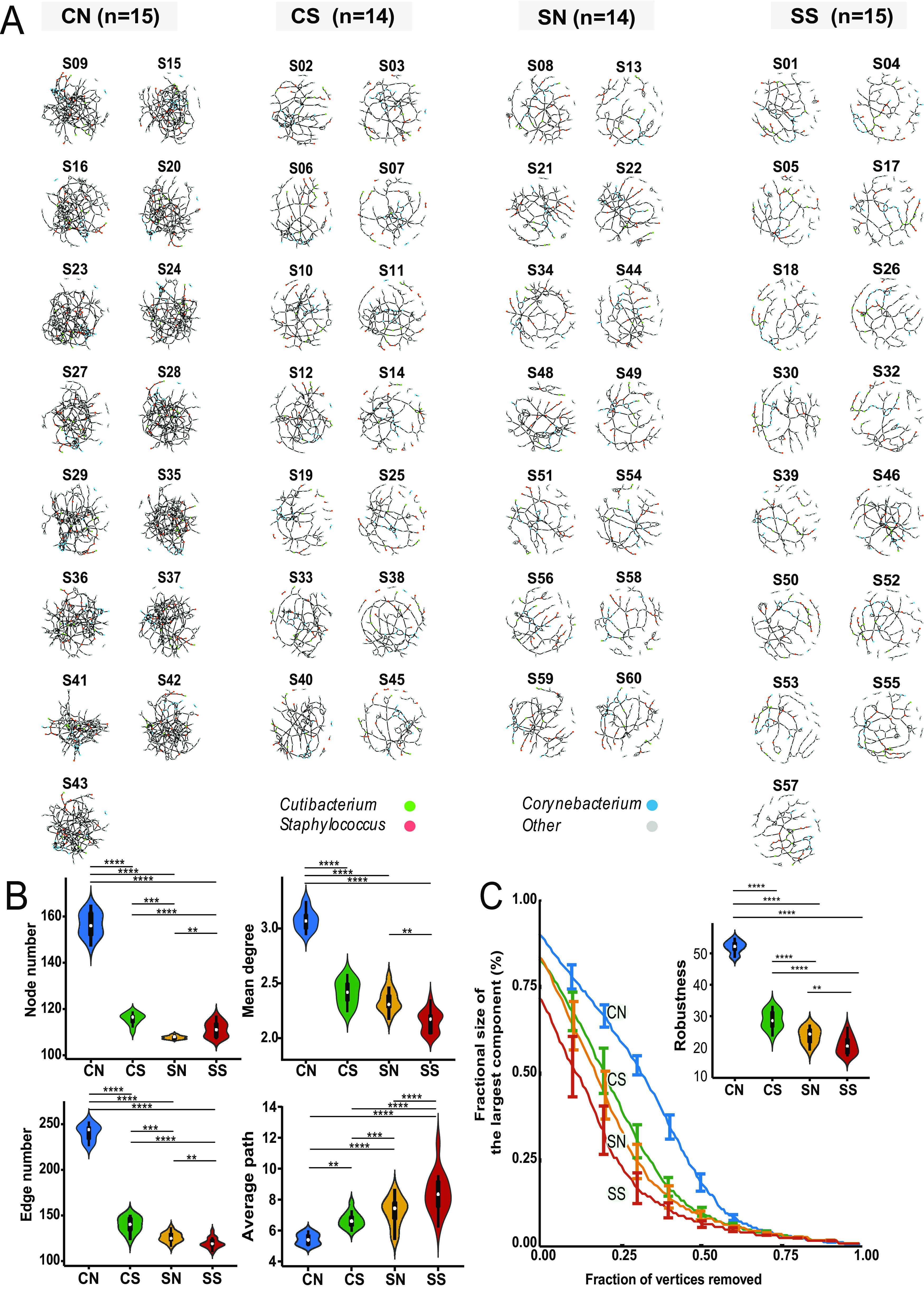
Analysis of the microbiome network of individual (MNI) for each subject under different pollution conditions. There were 58 MNIs corresponding to 58 subjects, respectively. (A) The MNI of each subject. Fifteen MNIs of the 15 subjects in the lowest-pollution group (CN) had the densest connections between the four groups. Three dominating genera (accounting for over 50% of the total abundance of all species), *Cutibacterium* (green), *Corynebacterium* (blue), and Staphylococcus (red), were colored. (B) Violin chart demonstrating the connectivity property of MNIs for each group of subjects, including node number, edge number, mean degree, and average path (Wilcox test *P* value: **, <0.05; ***, <0.001; ****, <0.0001). Clearly, the network connectivity decreased (CN > CS > SN > SS) with decreased pollution stress. (C) Line chart with error bars (median) demonstrated the fragility change as nodes were removed in each MNI network. The robustness of MNIs decreased (CN > CS > SN > SS) with decreased pollution stress and was consistent with the result of MNP. Both a larger area under the curve and a higher robustness value indicated the corresponding network was more robust.

While MNP analysis discloses the trend of microbiome network fragility with increasing pollution stress at a population level, it cannot reveal individual or heterogeneous features of a microbial community at an individual level. To analyze the differences of MNI between the four groups, we conducted principal-coordinate analysis (PCoA). We calculated Bray-Curtis distance of samples based on degree of nodes and distribution of each node (microbe) in MNI networks. We observed nonoverlapping distributed samples of each group (adjusted Rand index [ARI] = 1) which indicated that different network characteristics exist between four groups (Fig. S2). Thus, we further investigated the connectivity and fragility of the microbiome network using our MNI approach (see Materials and Methods for details), which also enabled statistical significance testing. MNIs for each subject were shown in Fig. 3A. The MNIs of CN subjects showed the largest connectivity between these four groups, which agreed with the trend of MNPs as shown in Fig. 2.

10.1128/mSystems.00319-21.2 FIG S2 PCoA based on MNIs of CN, CS, SN, and SS groups. Download FIG S2, PDF file, 0.1 MB. Copyright © 2021 Wang et al. 2021 Wang et al. https://creativecommons.org/licenses/by/4.0/ This content is distributed under the terms of the Creative Commons Attribution 4.0 International license.

Network attributes in terms of node number, edge number, mean degree, and average path were evaluated with a Wilcox test to further elucidate whether pollution stress affected skin MNIs. As illustrated in Fig. 3B, the groups could be ordered CN > CS > SN > SS in terms of node number of MNIs among these four groups and CN > CS > SN > SS in terms of both edge number and mean degree, while the reverse pattern, CN < CS < SN < SS, occurred in terms of the average path. All of the three network attributes indicated weaker network connectivity with increasing exposure to pollution stress. We also conducted analysis on the system collapse of MNIs to investigate microbiome fragility at an individual level (Fig. 3C). The robustness of MNIs showed significant difference between these four groups ordered as CN > CS > SN > SS, suggesting that pollution stress was associated with fragile skin MNIs or that MNIs became fragile under pollution stress. This result was also consistent with our findings on MNP (Fig. 2B and C).

### Skin microbiota mediated host response to pollution stress.

Our previous report demonstrated that urbanization-related pollution stress significantly affected skin physiological properties, including skin indices like total antioxidant capacity, cholesterol levels, vitamin E (VE), and transepidermal water loss (TEWL), and the activities of stratum corneum tryptic enzyme (SCTE) and catalase (18). While there is evidence indicating that skin microbes may be involved in certain biochemical events related to cholesterol esterification (21), skin antioxidant defense (22), and epidermal proteolytic activity (23), the mediative role of microbes in driving skin biophysical and biochemical responses toward environmental stimuli remains to be elucidated.

Here, we used a mediation model to evaluate whether or not the skin microbiome could mediate pollution’s effect on the six affected skin indices (Table S1). In a previous study (10), alpha diversity and network attributes of the soil microbiome were reported to play a key role in mediating plant-soil-microbe links. Thus, we collected 21 microbial properties, including 9 MNI variables and 12 alpha diversity variables, for mediation analysis. We obtained 6 (skin indices) × 21 (microbial properties) × 2 (binary directions), making a total of 252 models. We found 14 of these 252 models had significant mediation effects, as illustrated by the significance of the mediation index (Table S2A and B).

10.1128/mSystems.00319-21.4 TABLE S1 Essential variables (network properties, diversity attributes, and skin indices) in mediation analysis and all their models. Download Table S1, DOCX file, 0.04 MB. Copyright © 2021 Wang et al. 2021 Wang et al. https://creativecommons.org/licenses/by/4.0/ This content is distributed under the terms of the Creative Commons Attribution 4.0 International license.

10.1128/mSystems.00319-21.5 TABLE S2 Mediation analysis for three variables. (A) Illustration of mediation model. (B) Coefficient table of models that have significant mediation effect. Each model contains 3 variables (X, exposure; M, mediator; Y, outcome). Each number in the table represents the coefficient of each path (a, X to M; b, M to Y; c, X to Y; total, total effect a*b + c; mediation, mediation effect a*b). Red number indicates the significant coefficient with a negative value, black number indicates the significant coefficient with a positive value, and gray number indicates the nonsignificant coefficient. (C) Coefficient table of all mediation models, including nonsignificant models. Download Table S2, XLSX file, 0.2 MB. Copyright © 2021 Wang et al. 2021 Wang et al. https://creativecommons.org/licenses/by/4.0/ This content is distributed under the terms of the Creative Commons Attribution 4.0 International license.

**FIG 4:**
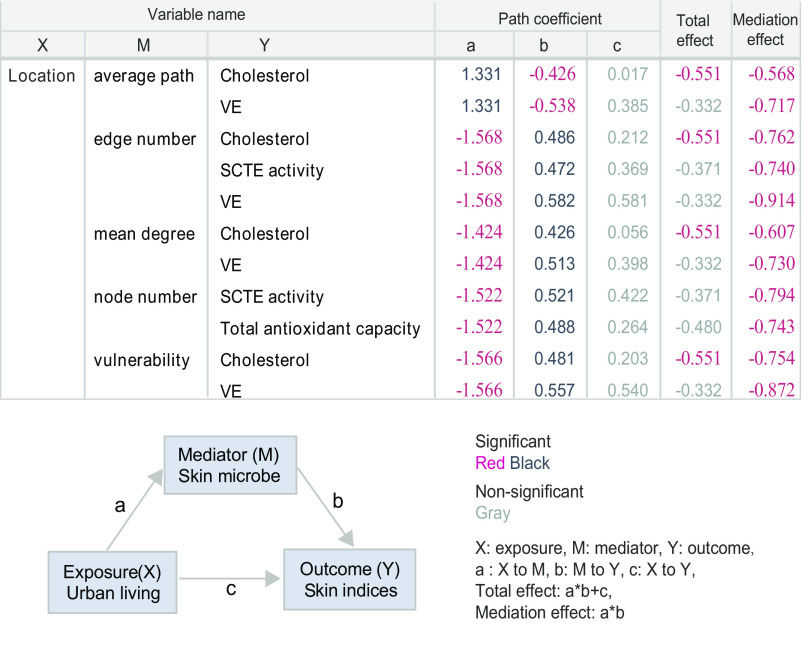
Mediation analysis reveals the mediation role of the skin microbiome. Only models that had consistent mediation effects are contained in the table. Each model contains three variables (X, exposure; M, mediator; and Y, outcome). Each number in the table represents the coefficient of each path (a, X to M; b, M to Y; c, X to Y; total, total effect a*b+c; mediation, mediation effect a*b). Red numbers indicate significant coefficients with a negative value, black numbers indicate significant coefficients with a positive value, and gray numbers indicate nonsignificant coefficients.

We found that alpha diversity had no significant mediating effect in all of our models, while multiple MNI attributes of connectivity (edge number, node number, mean degree, and average path) and fragility (robustness) had significant mediating effects on cholesterol, total antioxidant capacity, VE, and SCTE activity (Fig. 4). For example, cholesterol and VE levels of the skin were reduced due to decreased MNI connectivity and robustness upon pollution exposure. Notably, in reverse models where skin indices were set as a mediation variable from regional air pollution exposure to MNI properties, the mediation effects of skin attributes of cholesterol, total antioxidant capacity, VE, and SCTE were insignificant, while TEWL showed bidirectional mediation effects (Table S2A and B). The result of our mediation analysis indicated that fragile MNIs mediated the adverse effects of air pollution on skin attributes, thus damaging skin health.

### A smoking lifestyle deepened the negative effects of pollution stress on skin microbiota.

**FIG 5:**
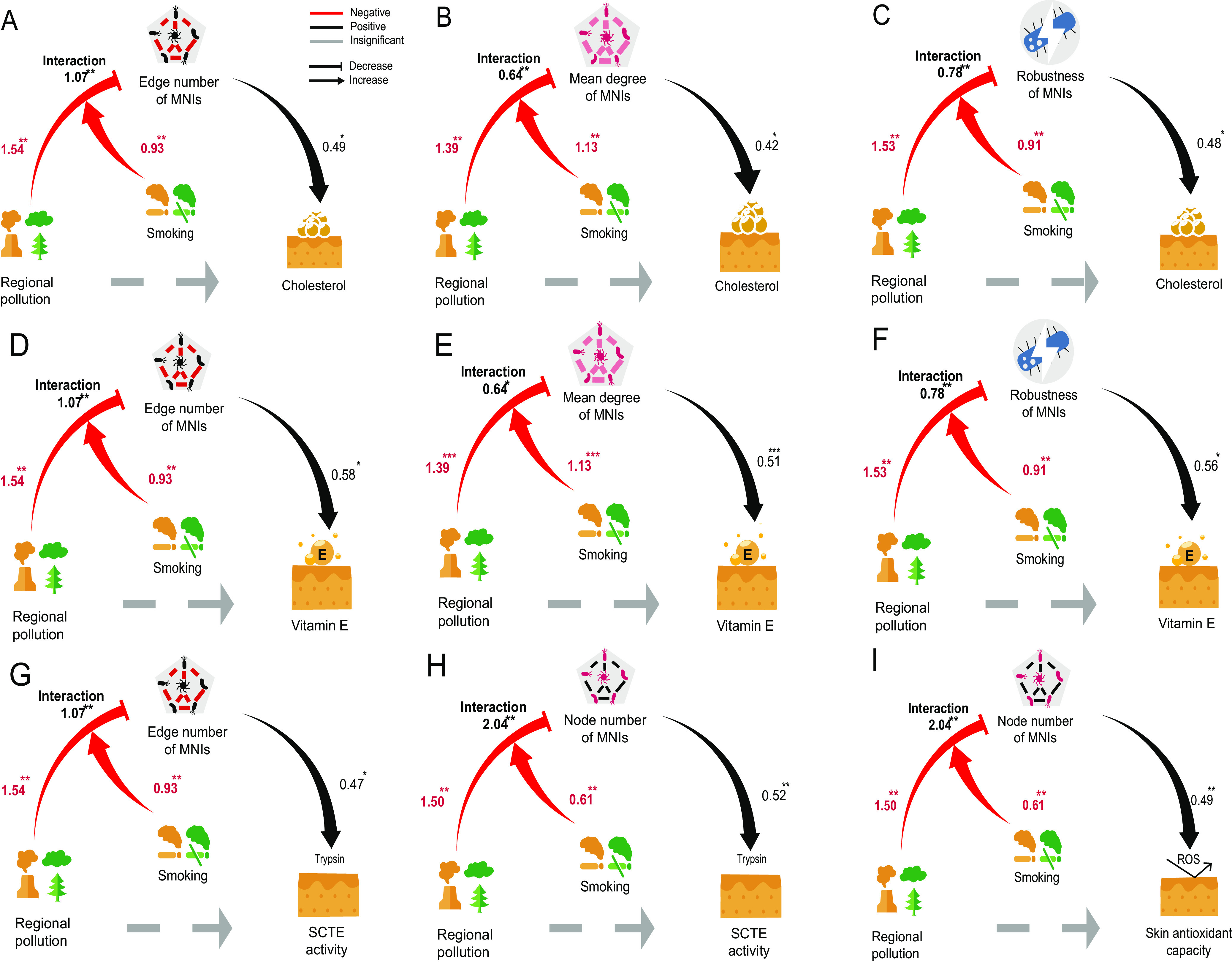
Moderation analysis with conditional process analysis linking pollution and microbiome network properties to skin attributes. (A to I) In each model, regional air pollution was a mediator, and smoking was a moderator. MNIs can mediate the damage of pollution on skin indices, while smoking can moderate the path between pollution and MNI properties. As a result, we obtained multiple models that were biologically plausible and were also statistically significant, tested by conditional process analysis (moderated mediation). Here, the number near the path was path coefficient, which was labeled with *P* value significance levels (*, <0.05; **, <0.001; ***, 0.0001).

Cigarette smoke is another source of pollution stressors known to affect skin health (24). Certain bacteria of skin origin were reported to display a metabolic capacity to break down organic pollutants, like benzo(a)pyrene, that exist in both cigarette smoke and automobile exhaust fumes (25), suggesting a potential combinatorial effect of regional air pollution and a smoking lifestyle on the skin microbiome. Indeed, our MNI results indicated that Shanghai smokers (SS) had the least robust microbiome network compared with Chongming nonsmokers (CN). To further quantify the moderating effect of smoking on a model path from exposure (regional air pollution) to mediation variables (MNI property), by conditional process analysis (CPA), we conducted a moderation analysis of these 11 models as shown in Fig. 4, 9 of which were statistically significant (Table S3), as shown in Fig. 5.

10.1128/mSystems.00319-21.6 TABLE S3 Conditional process analysis for four variables. (A) Illustration of conditional process model. (B) Coefficient table of models that have significant moderation effect. Download Table S3, PDF file, 0.1 MB. Copyright © 2021 Wang et al. 2021 Wang et al. https://creativecommons.org/licenses/by/4.0/ This content is distributed under the terms of the Creative Commons Attribution 4.0 International license.

On the path from exposure (regional air pollution) to mediation variables (MNI properties), the moderation effect of smoking was consistent across all nine models (interaction between smoking and pollution path coefficient β < 0, *P* < 0.05); namely, a smoking lifestyle deepened the negative effect of pollution on MNI properties, including edge number, node number, mean degree, and robustness, which then mediated skin physiological properties. The model with the highest *R*^2^ value was the urban (smoking)-edge number-cholesterol model (Fig. 5B, conditional process analysis, *R*^2^ = 0.1656). This model indicated that pollution reduces the edge number of the microbiome network/community, and then, such a fragile microbiome community results in less cholesterol on the skin surface. Moderation analysis using a conditional process model shows that smoking significantly moderated (path coefficient β = 1.066, *P* < 0.0001) (Table S3) the effect or interaction from regional air pollution on the robustness of MNIs, thus deepening the negative effects of pollution stress on skin microbiota. This result indicated that smoking and regional air pollution actually had a combinatorial effect to reduce microbial interactions, leading to the decrease of cholesterol and VE levels, as well as reduced SCTE and the total antioxidant capacity on the skin surface.

## DISCUSSION

The human microbiome is comprised of numerous microbial taxa, each of which potentially has interactions with many other taxa. Elucidating microbial interactions should help uncover the functional importance of the microbiome in health and disease. In this study, we conducted a network-based investigation to elucidate the characteristics of a skin microbiome network/community at an individual level and to quantify the mediation and further moderation effects of a skin microbial community on the skin’s physiological response toward pollution stressors. To better control heterogenous factors such as subject backgrounds, climate condition, and socioeconomic status that may affect the skin and the skin microbiome, we conducted the study by comparing male taxi drivers from two neighboring areas with different pollution levels with a desired contrast by controlling possible confounding factors such as subjects’ age, years of working, and service life of the taxi to investigate the effect of pollution stress on skin microbiome. This was the first study on the mediation effect of the microbiome by the use of mediation analysis and conditional process analysis at an individual resolution. This work represents a step toward deciphering the importance of the microbiome in mediating the directional effect of pollution on skin health. Our data revealed for the first time that MNIs significantly mediate the change of cholesterol, antioxidant capacity, VE, TEWL, and SCTE activity of the skin resulting from pollution stress, while a smoking lifestyle amplifies the damaging effect of air pollution on the microbiome and the skin. In contrast, most of the tested skin indices, except for TEWL, did not exert significant mediation effects on MNI properties. This suggests that in our data set, location-related microbial changes are independent of skin indicators (26). Notably, we found that network properties of MNIs, rather than the traditional alpha diversity properties, played a mediation role in our models. This observation underlies the importance of microbial interactions for skin indices. Moreover, the effect of pollution on MNIs was additive to lifestyle factors (e.g., smoking). The current study was restricted to the microbiome of facial skin (upper cheek) regions. Given that the skin microbiome is diverse across different body sites, further investigation into the mediation effect of the skin microbiome across multiple body sites could help to provide a more holistic view on microbiome functions related to skin health.

The skin microbiome is regarded as an integral part of the skin’s natural defense system against external stressors (20). By mediation analysis, we found that the skin microbiota mediated several aspects of the stratum corneum (SC) function under pollution stress. Cholesterol is among the key component of the SC intercellular lipid layer (27). SCTE is known as kallikrein-related peptidase 5 (KLK5), and its activity regulates SC desquamation to maintain the skin barrier function (28). Our data indicated that pollution stress may affect skin barrier function via altering the skin microbiome network. Dominant skin bacteria, such as Cutibacterium acnes and Staphylococcus epidermidis, were reported to modify skin lipids by esterification of cholesterol to cholesteryl esters, which may affect SC structure (21, 29). Skin enzyme activities can also be regulated by skin microbiota via regulating skin pH through hydrolyzing skin lipids to free fatty acids (30). These insights support a possible mediation role of skin microbiota in modulating skin barrier function.

In addition to the barrier function of the skin, SC supports the skin’s innate antioxidant system (31). We found that a fragile microbiome network resulting from pollution stress can alter the skin’s total antioxidant capacity and the level of VE, one of the skin antioxidants (32). The disrupted antioxidant system may further lead to skin inflammation and disrupted barrier function (33).

In our model, TEWL was the only skin index that showed bidirectional effects, whereby pollution stress affected TEWL via the skin microbiome and vice versa. Biologically, TEWL is a key attribute of skin barrier integrity. An increased level of TEWL indicates an altered skin hydration and worsened skin barrier function. It may be possible that a fragile skin microbiome network resulting from pollution stress disturbs skin barrier integrity, leading to the increased TEWL levels. Conversely, pollution exposure may lead to increased TEWL, which impacts skin hydration, a key attribute of the skin that affects the skin microbiome community (20).

Compared with traditional network methods, the advantage of our MNI method is that it is able to reveal previously hidden features, such as network biomarkers and “dark” microbes/species, based on its sample-specific nature. A network biomarker (12, 13) is a biomarker measured by the interaction or correlation between two species, rather than their concentrations or abundances (traditional molecular biomarkers), and a “dark” species is one where there was no differential expression of this species but there is differential interaction/correlation with this species (11, 14). Although a structural equation model has been applied to the analysis of the microbiome network of population (MNP) previously (10), it requires the assignment of a set of samples to the same attributes of the MNP. In contrast, an MNI can represent each person’s characteristics at a network level. In our data set as shown in Fig. 2, the average path of MNP displayed inconsistent trends between the four groups (SS < CN < CS < SN). However, by MNI as shown in Fig. 3, a consistent trend can be identified (CN < CS < SN < SS). Moreover, the partial correlation generated by MNI was a direct association reflecting the microbial interaction or the underlying structure of microbial communities (34), which eliminated the indirect associations (spurious relations) generated by traditional single-sample network methods (12, 16, 35). To further validate our finding, we analyzed an independent cohort for the skin microbiome upon pollution from Dalian (low pollution) and Baoding (high pollution) cities in China (see Fig. S3 in the supplemental material). In this data set, we observed the same trend of reduced network connectivity in pollution (Dalian > Baoding) as of our cohort (Chongming > Shanghai). Our MNI algorithm enabled us to investigate network rewirings, to help in revealing “dark” microbes whose interaction cannot be detected by traditional microbiome abundance analysis, to study the mediation effect, and further to identify network biomarkers at an individual level. We anticipate that the MNI method will serve as a backbone for precision medicine based on the microbiome and allow exploration of new hypotheses on its relevance to environmental factors and human health. We propose that the MNI properties could serve as a novel network biomarker (11, 12, 14) of skin health.

10.1128/mSystems.00319-21.3 FIG S3 Analysis of the microbiome network of individual (MNI) from cheek microbiome data reported by Leung et al. (41). In total, 85 MNIs were constructed corresponding to 47 subjects from Dalian (low pollution) city and 38 subjects from Baoding (high pollution) city in China. Each node represented a microbial taxon (OTU). (a) Forty-seven MNIs of the low-pollution group (Dalian) visually showed higher connections than 38 MNIs of the high-pollution group (Baoding). (b to e) Each violin chart demonstrated the connectivity property of MNIs for each group of subjects, including edge number (b), node number (c), mean degree (d), and robustness (e) (Wilcox test *P* value: **, <0.05; ****, <0.0001). MNI connectivity decreased with the pollution stress (Dalian > Baoding). Download FIG S3, PDF file, 4.0 MB. Copyright © 2021 Wang et al. 2021 Wang et al. https://creativecommons.org/licenses/by/4.0/ This content is distributed under the terms of the Creative Commons Attribution 4.0 International license.

### Conclusion.

This is the first study that describes the mediation effect of the skin microbiome on the damage effect of environmental factors on skin health. We developed the microbiome network of an individual (MNI), to analyze what effect of pollution stressors exerted on the microbiome network and how rewiring of the microbiome network mediates downstream skin responses toward pollution stressors. We provide evidence here that the skin microbiome can act as the first interface of the body responding to environmental stress. The stability and integrity of the skin microbial community can play a critical mediation role that regulates skin biophysical and biochemical properties. Improving the skin microbial community stability may be a sensible strategy for delivering skin benefits.

## MATERIALS AND METHODS

### Clinical study design and sampling.

The air quality and urbanization data were obtained from the 2014, 2015, and 2016 Shanghai Municipal Bureau of Ecology and Environment Shanghai Environment Yearbook. Middle-aged (mean age 43 years, SD = 7, ranging from 30 to 55 years) healthy male taxi drivers from rural CM and urban SH were recruited, each of whom had been working at the current place for at least 5 years. In total, 30 SH subjects and 30 CM subjects, with half smokers and half nonsmokers in each group, were analyzed. A method of stratification was used to ensure a balance of age, years of working, and service life of the taxi between each group to control potential confounding variables that may affect skin microbiome. Forty-three, 11, and 2 years were the median value of the corresponding variables of age, years of working, and service life of the taxi. Detailed information on this clinical study was previously reported (18). In brief, exclusion criteria included daily alcohol drinking, skin diseases, regular medication with antihistamines or anti-inflammatory drugs, or having had facial cosmetic procedures. Informed consent was obtained from all subjects, with the study protocol being reviewed and approved by the Independent Ethics Committee at the Shanghai Clinical Research Center. All subjects were asked to refrain from washing their faces with cleansers before the study visit for at least 12 h since the last face wash. On the day of the visit, subjects were asked not to wear any skin care products on the face. After 1 h of equilibrium period on site, TEWL (transepidermal water loss) (Tewameter TM300) and cheek hydration (Corneometer CM825) were measured according to well-established industrial practice. All instrumental measures were repeated, and the average data were used for data analysis. After instrumental measurement, standard D-Squame tape stripping (Cuderm, Dallas, TX, USA) was conducted on the upper right cheek of each subject 13 consecutive times with all tapes individually stored in a 96-well sample collection box at −80°C. Sebutapes (Cuderm, Dallas, TX) were used for sebum collection on the central forehead area. Immediately after sample collection, all the Sebutapes were stored at −80°C as well until subjected to extraction for endpoint analyses. The first layer of D-Squame tape was used for microbiome 16S amplicon sequencing, and the fifth layer of the tape was used to measure SCTE, total antioxidant capacity (TAOC), and catalase activity. Sebutapes were used to measure the levels of cholesterol and vitamin E.

### 16S rRNA sequencing using Illumina MiSeq PE300.

The first layer of D-Squame tape stripping samples was used for microbiome analysis. DNA was extracted from the samples using a DNA extraction kit (DNeasy blood and tissue kit, catalog no. 69506; Qiagen, Hilden, Germany) following the manufacturer’s instructions. The microbial DNAs were sequenced at the variable region (V1 to V3) of the 16S rRNA gene for bacterial classification. The V1 to V3 region was amplified using a primer set (forward primer, AGAGTTTGATYMTGGCTCAG, and reverse primer, ATTACCGCGGCTGCTGG) and sequenced by Beijing Genomics Institute (BGI; Wuhan, China) by using fusion primers with dual indices and adapters. The quantity and quality of the libraries were analyzed by Bioanalyzer (Agilent Technologies, CA, USA). Only qualified libraries were processed and analyzed via Illumina MiSeq PE300 (pair-end) sequencing, initially resulting in approximately 21.9 million raw sequence paired reads. After quality processing, 20.0 million overlapping contigs were produced after Pandaseq 2.9 and grouped into 486 OTUs.

### Skin index analysis.

Analysis of skin indices was previously reported (18). In brief, the fifth layer of the D-Squame tape stripping samples was extracted with phosphate-buffered saline (PBS) containing 0.3% Triton X-100 and subjected to catalase activity, total antioxidant capacity (TAOC), and stratum corneum tryptic enzyme (SCTE) measurements as previously reported (18). Sebutapes were extracted individually using 5 ml of hexane, under three times of 5-min sonification. After that, the extract was dried with rotary evaporation (9 kPa, 35°C) before being reconstituted with 2 ml of hexane for gas chromatography-mass spectrometry (GC-MS) analysis. Reference compounds were used in GC-MS as reference for quantification. In total, cholesterol, vitamin E, and squalene were quantified for each Sebutape. Among them, squalene content was used as normalization reference (18).

### Data normalization.

Data normalization of skin attributes and MNI attributes was performed with Yeo-Johnson normalization using the yeojohnson function (R package bestNormalize).

### Network connectivity and fragility calculations.

The connectivity of a network is an important indicator of network complexity. To measure network connectivity, we calculated various network attributes, such as node (vertex) number, edge (link) number, mean degree, connection component, and average path using the R package igraph. The node number represents the sum of all nodes in a network, and the edge number represents all significant partial correlations (SPIEC-EASI) between nodes in a network. The degree of a node is the number of edges connecting the given node. The mean degree is the mean of degrees for all nodes, and the average path is the mean length of the shortest paths of all the nodes. The connected component in a network is a subnetwork where any two nodes are connected by at least a path. When the node number, edge number, and mean degree are larger, a network has a higher connectivity. When the average path is lower, a network has higher connectivity.

To measure network fragility, we simulated the ecological disruption under the deletion of the species. Given the *P* OTUs in a network, each OTU was removed one by one in a random order. The fractional size of the largest component statistics (*y* axis in Fig. 2C) was defined as the ratio of the node number in the largest connected component to the total node number in the network, and the ratio was recalculated when each node was removed. The percentage of removed nodes was taken as the *x* axis in Fig. 3C. To ensure reliable results, we ran the above process to remove the nodes 1,000 times. To plot the robust curve, the median of the fractional size of the largest component statistics in the 1,000 curves was taken as the *y* coordinates for the curve. The robustness value *R* of a network was defined as a function σ which was based on the decreasing order of the fractional size of the largest component statistics as nodes were removed (5). The robustness value of a network was the median of the robustness value of the 1,000 curves.

(1) $$R=\frac{1}{P}\sum_{i=1}^{P}\sigma (i/P)$$

where *i* represents *i*th OTU. A larger area under the curve and higher robustness value of the network indicate a less fragile network.

### Microbial association network (MNP and MNI) construction.

Before network construction, we removed OTUs that had frequencies of less than 65% (99% in the validation cohort with more sparse sequence) in all samples of each group. Then, we performed SPIEC-EASI to infer a microbial association network, using the neighborhood selection (mb) method with a minimum λ threshold of 0.01 (18). All steps were computed using the R package SpiecEasi (version 1.0.7). The microbiome network of population (MNP) with *N* subjects was constructed directly using SpiecEasi. The MNI method was used to obtain the microbiome network of individual (MNI) for each subject as described below.

### MNI method and algorithm.

Our MNI method was proposed to investigate the network characteristics of each subject in a single-sample network manner as shown in Fig. 1. Aitchison indicated that 16S sequencing data were compositional and constrained to a simple dimension (sum to 1), therefore suffering from the problem of serious spurious correlation (17), if we simply use the traditional correlation methods. The log ratio of the unobserved absolute species abundance was equivalent to the log ratio of a compositional species abundance (17):

(2) $$\log(\frac{x}{y})=\log(\frac{\omega_{x}/m}{\omega_{y}/m})=\log(\frac{\omega_{x}}{\omega_{y}})$$

where *x* and *y* are the relative abundances of the two different species, *m* is total absolute abundances of all species within this sample, and ω*_x_* and ω*_y_* are the absolute abundances of those two species. The log ratio $\log(\frac{x}{y})$ is the basis for studying compositional data, rather than the original *x* and *y*.

Then, to eliminate spurious correlation in compositional data, Ω = *Cov*[log ω], the covariance matrix of the log-transformed absolute abundance *ω*, can be approximated via reference 17:

(3) Γ=GΩG

where *ω* is the absolute abundance of each species, $G=I_{p}-\frac{1}{p}J$, *I_p_* is the *p*-dimensional identity matrix, *J* is the *p*-dimensional all-ones vector, and Γ is $\mathrm{Cov}[\log\frac{x}{y}]$. Therefore, Ω^−1^ is the inverse covariance of the underlying unmeasured absolute basis, corresponding to the precision matrix and partial correlation (36). The resulting matrix Ω^−1^ can estimate an inferred network, and the elements in Ω^−1^ are the partial correlations of the population network for *p* species with *N* samples/subjects.

In this work, instead of a population network with *N* samples, we further derived a sample-specific network by sample-specific partial correlation, based on the fact that the partial correlation of a population network is a linear combination of each sample (19), i.e.,

(4) $$r_{\mathrm{xy},z}^{\left(N\right)}=\frac{N\sum_{i}^{N}e_{\mathrm{xz}}^{\left(i\right)}e_{\mathrm{yz}}^{\left(i\right)}}{\sqrt{N\sum_{i}^{N}e_{\mathrm{xz}}^{{\left(i\right)}^{2}}}\sqrt{N\sum_{i}^{N}e_{\mathrm{yz}}^{{\left(i\right)}^{2}}}}$$

where $r_{\mathrm{xy},z}^{\left(N\right)}$ is the partial correlation between *x* and *y* conditioned on *z* with all *N* samples, and $e_{\mathrm{xz}}^{\left(i\right)}=x^{\left(i\right)}-b_{\mathrm{xz}}Z^{\left(i\right)}$ and $e_{\mathrm{yz}}^{\left(i\right)}=y^{\left(i\right)}-b_{\mathrm{yz}}Z^{\left(i\right)}$ are the *i*th elements of residuals *e_xz_* and *e_yz_* (37), respectively. *b_xz_* and *b_yz_* are regression coefficients corresponding to *e_xz_* and *e_yz_*, respectively, while *x_i_*, *y_i_*, and *z_i_* are the relative abundance of species *x*, *y*, and *z* in the *i*th sample. Actually, provided that the relative abundances of *N* samples are given, SPIEC-EASI gives the precision matrix, which was proportional to the partial correlation matrix. In this work, we directly used its result as an approximate partial correlation.

Based on the single-sample method developed by Kuijjer (16), we can represent a partial correlation for a single sample (i.e., the *q*th sample) as follows by considering that each sample contributes equally to a partial correlation of population with *N* samples:

(5) $$l_{\mathrm{xy},z}^{\left(q\right)}=Nr_{\mathrm{xy},z}^{\left(N\right)}-(N-1)r_{\mathrm{xy},z}^{\left(N/q\right)}$$

This means that the partial correlation of species *x* and *y* in a given *q*th sample $l_{\mathrm{xy},z}^{\left(q\right)}$ is equal to the difference between a partial correlation using all *N* samples $r_{\mathrm{xy},z}^{\left(N\right)}$ and a partial correlation using all except for this given sample $r_{\mathrm{xy},z}^{\left(N/q\right)}$. Here, $r_{\mathrm{xy},z}^{\left(N\right)}$ is the partial correlation between *x* and *y* conditioned on *z* with all *N* samples, and $r_{\mathrm{xy},z}^{\left(N/q\right)}$ is the partial correlation between *x* and *y* conditioned on *z* with all samples except the *q*th sample.

We can transform the partial correlation of equation 4 into

(6) $$l_{\mathrm{xy},z}^{\left(N\right)}=\frac{\frac{\sum_{i}^{N}x_{i}y_{i}}{N-1}-b_{\mathrm{yz}}\frac{\sum_{i}^{N}x_{i}z_{i}}{N-1}}{\sqrt{\left(\frac{\sum_{i}^{N}x_{i}^{2}}{N-1}-\frac{b_{\mathrm{xz}}\sum_{i}^{N}x_{i}z_{i}}{N-1})(\frac{\sum_{i}^{N}y_{i}^{2}}{N-1}-\frac{b_{\mathrm{yz}}\sum_{i}^{N}y_{i}z_{i}}{N-1}\right)}}=\frac{\bar{\mathrm{xy}}-b_{\mathrm{yz}}\bar{\mathrm{xz}}}{\sqrt{\left(\bar{x^{2}}-b_{\mathrm{xz}}\bar{\mathrm{xz}})(\bar{y^{2}}-b_{\mathrm{yz}}\bar{\mathrm{xz}}\right)}}$$

(7) $$\begin{array}{l} l_{\mathrm{xy},z}^{\left(N/q\right)}=\frac{\frac{\sum_{i\;\neq q}^{N}x_{i}y_{i}}{N-2}-b_{\mathrm{yz}}\frac{\sum_{i\;\neq q}^{N}x_{i}z_{i}}{N-2}}{\sqrt{\left(\frac{\sum_{i\;\neq q}^{N}x_{i}^{2}}{N-2}-\frac{b_{\mathrm{xz}}\sum_{i\;\neq q}^{N}x_{i}z_{i}}{N-2})(\frac{\sum_{i\;\neq q}^{N}y_{i}^{2}}{N-2}-\frac{b_{\mathrm{yz}}\sum_{i\;\neq q}^{N}y_{i}z_{i}}{N-2}\right)}}\\ =\frac{\bar{x^{\left(N/q\right)}y^{\left(N/q\right)}}-b_{\mathrm{yz}}\bar{x^{\left(N/q\right)}z^{\left(N/q\right)}}}{\sqrt{\left(\bar{x^{{\left(N/q\right)}^{2}}}-b_{\mathrm{xz}}\bar{x^{\left(N/q\right)}z^{\left(N/q\right)}})(\bar{y^{{\left(N/q\right)}^{2}}}-b_{\mathrm{yz}}\bar{x^{\left(N/q\right)}z^{\left(N/q\right)}}\right)}} \end{array}$$

For a sufficiently large value of *N*, we can easily show $\bar{x^{\left(n/q\right)}}\to \bar{x^{\left(n\right)}}$ (16); therefore, $\bar{x^{{\left(n/q\right)}^{2}}}\to \bar{x^{{\left(n\right)}^{2}}},\;\bar{y^{{\left(n/q\right)}^{2}}}\to \bar{y^{{\left(n\right)}^{2}}},\;\bar{x^{\left(n/q\right)}z^{\left(n/q\right)}}\to \bar{x^{(n)z^{\left(n\right)}}}$. Then

(8) $$\begin{array}{l} l_{\mathrm{xy},z}^{\left(q\right)}=Nr_{\mathrm{xy},z}^{\left(n\right)}-(N-1)r_{\mathrm{xy},z}^{\left(n/q\right)}\\ \;=N\frac{\bar{\mathrm{xy}}-b_{\mathrm{yz}}\bar{\mathrm{xz}}}{\sqrt{\left(\bar{x^{2}}-b_{\mathrm{xz}}\bar{\mathrm{xz}})(\bar{y^{2}}-b_{\mathrm{yz}}\bar{\mathrm{xz}}\right)}}-(N-1)\frac{\bar{x^{\left(N/q\right)}y^{\left(N/q\right)}}-b_{\mathrm{yz}}\bar{x^{\left(N/q\right)}z^{\left(N/q\right)}}}{\sqrt{\left(\bar{x^{{\left(N/q\right)}^{2}}}-b_{\mathrm{xz}}\bar{x^{\left(N/q\right)}z^{\left(N/q\right)}})(\bar{y^{{\left(N/q\right)}^{2}}}-b_{\mathrm{yz}}\bar{x^{\left(N/q\right)}z^{\left(N/q\right)}}\right)}} \end{array}$$

In the limit of a sufficiently large *N*, $\frac{N}{N-1}$ and $\frac{N-1}{N-2}$ are approximately equal to 1. Thus, the equation above can be simplified as follows:

(9) $$\begin{array}{l} \underset{N\to \infty }{\lim}l_{\mathrm{xy}}^{\left(q\right)}=\underset{N\to \infty }{\lim}[\frac{\frac{N\sum_{i}^{N}x_{i}y_{i}}{N-1}-\frac{Nb_{\mathrm{yz}}\sum_{i}^{N}x_{i}z_{i}}{N-1}}{\sqrt{\left(\bar{x^{2}}-b_{\mathrm{xz}}\bar{\mathrm{xz}})(\bar{y^{2}}-b_{\mathrm{yz}}\bar{\mathrm{xz}}\right)}}\\ -\frac{\frac{(N-1)\sum_{i\;\neq q}^{N}x_{i}y_{i}}{N-2}-\frac{(N-1)b_{\mathrm{yz}}\sum_{i\;\neq q}^{N}x_{i}z_{i}}{N-2}}{\sqrt{\left(\bar{x^{{\left(N/q\right)}^{2}}}-b_{\mathrm{xz}}\bar{x^{\left(N/q\right)}z^{\left(N/q\right)}})(\bar{y^{{\left(N/q\right)}^{2}}}-b_{\mathrm{yz}}\bar{x^{\left(N/q\right)}z^{\left(N/q\right)}}\right)}}]\\ =\frac{\frac{x_{i}y_{i}}{N}-b_{\mathrm{yz}}\frac{x_{i}z_{i}}{N}}{\sqrt{\left(\bar{x^{2}}-b_{\mathrm{xz}}\bar{\mathrm{xz}})(\bar{y^{2}}-b_{\mathrm{yz}}\bar{\mathrm{xz}}\right)}} \end{array}$$

Therefore,

(10) $$\underset{N\to \infty }{\lim}\frac{1}{N}\sum_{i}^{N}l_{\mathrm{xy}}^{\left(q\right)}=\frac{\sum_{i}^{N}\frac{x_{i}y_{i}}{N}-b_{\mathrm{yz}}\sum_{i}^{N}\frac{x_{i}z_{i}}{N}}{\sqrt{\left(\bar{x^{2}}-b_{\mathrm{xz}}\bar{\mathrm{xz}})(\bar{y^{2}}-b_{\mathrm{yz}}\bar{\mathrm{xz}}\right)}}$$

which is equal to $r_{\mathrm{xy},z}^{\left(N\right)}$ according to equation 5. Thus, the MNI algorithm is given as follows.

Step 1. Input the sequencing data (relative abundances of species: *x*, *y*, *z*, …) with *N* samples of *p* species. Let *q* = 1.
Step 2. Calculate each partial correlation $r_{\mathrm{xy},z}^{\left(N\right)}$ with *N* samples using SPIEC-EASI (equation 3).
Step 3. Calculate each partial correlation $r_{\mathrm{xy},z}^{\left(N/q\right)}$ with *N* – 1 samples by removing the *q*th sample using SPIEC-EASI (equation 6).
Step 4. By equation 8, calculate each sample’s specific partial correlation for each pair of species *x* and *y*.
Step 5. Let *q* = *q* + 1, and go to step 3 until *q* = *N*. Partial correlations of all pairs for each sample form a sample-specific network, or the microbiome network of an individual (MNI).

To illustrate the differences of MNI between the four groups, we conducted PCoA (R package vegan) based on Bray-Curtis distance of degree distribution of each node (microbes) in MNI networks. The degree matrix represents the microbial network characteristics from the perspective of nodes. Through PCoA based on degree matrix, we can visualize the difference of network characteristics between four groups.

### Mediation analysis and moderation analysis with a conditional process model.

Mediation analysis in this study was adopted from the well-established principle in the literature to quantify mediation effects (26, 38). We implemented our custom pipeline based on the R package Lavaan with the following two steps.

### (i) Mediation step.

To test our hypothesis on the direction, we did a two-directional analysis. In the first direction, we tested whether skin indices mediated the damage of urban stressors on the skin microbiome. In the second direction, we tested whether the skin microbiome mediated (path a*b) the damage of urban stressors on skin indices. As the result, we found that only the mediation paths through the skin microbiome (path a*b) were significant.

Specifically, for a given skin index, we tested the mediation effect of every microbial property, such as connectivity and robustness. Each mediation model was composed of three variables: regional pollution (dichotomous) as exposure, microbial property variables (or microbiome network) as mediation variables, and the skin index as an outcome. A total of 252 models, including 6 (skin indices) × 21 (microbial properties) × 2 (opposite directions) were tested. Finally, we obtained 11 models that had one-directional significant mediation effect coefficients (*P* < 0.05) among all 252 models (Fig. 4). One direction means that these models have an effect from the microbiome to the skin indices, and the skin indices have no effect on the microbiome. Note that we aimed to reveal how a microbiome network of a subject played a mediation role between air pollution and skin health, in this mediation step (mediation analysis).

### (ii) Moderation step.

Considering the potential reverse effect of the skin index on microbial properties, we also tested a model of regional pollution (dichotomous) as exposure, with the skin index as a mediation variable, and a microbial property variable as the outcome, using conditional process analysis. We found the models based on reverse effects of skin index on microbial property were all insignificant (mediation effect *P* > 0.05). Based on our 11 models, we quantified the moderating effect of smoking (dichotomous variable) on the path (air pollution to microbial property), by using a conditional process model in SPSS Process. Nine of the 11 models had a significant moderating effect (*P* < 0.05). Note that we aimed to identify how smoking affects or changes the interaction or path between air pollution and a microbiome network for each subject, in this moderation step (moderation analysis).

### Statistical analysis.

Alpha diversity (Shannon entropy) and beta diversity were generated by QIIME (39) and phyloseq (40). Beta diversity was visualized using a Bray-Curtis dissimilarity with nonmetric multidimensional scaling. To compare the alpha diversities between pollution levels, we performed a Kruskal-Wallis test for multiple groups, a Wilcoxon test for two groups, and *post hoc* correction. We used the Kruskal.test function and wilcox.test function in R. Analysis of variance between CS, CN, SS, and SN was performed using permutation analysis of variance (PERMANOVA) on the Bray-Curtis dissimilarity matrix. Analyses were performed using the R packages Vegan, Party, and HMP with *q* value to control for false discovery. ARI (adjusted Rand index) was calculated with adjustedRandIndex function in mclust package.
