# Supplementary figures and images for: Facial Skin Microbiota-Mediated Host Response to Pollution Stress Revealed by Microbiome Networks of Individual
Source: mSystems. 2021 Jul 27;6(4):e00319-21. doi: 10.1128/mSystems.00319-21 (PMC8407115; doi:10.1128/mSystems.00319-21)

**A**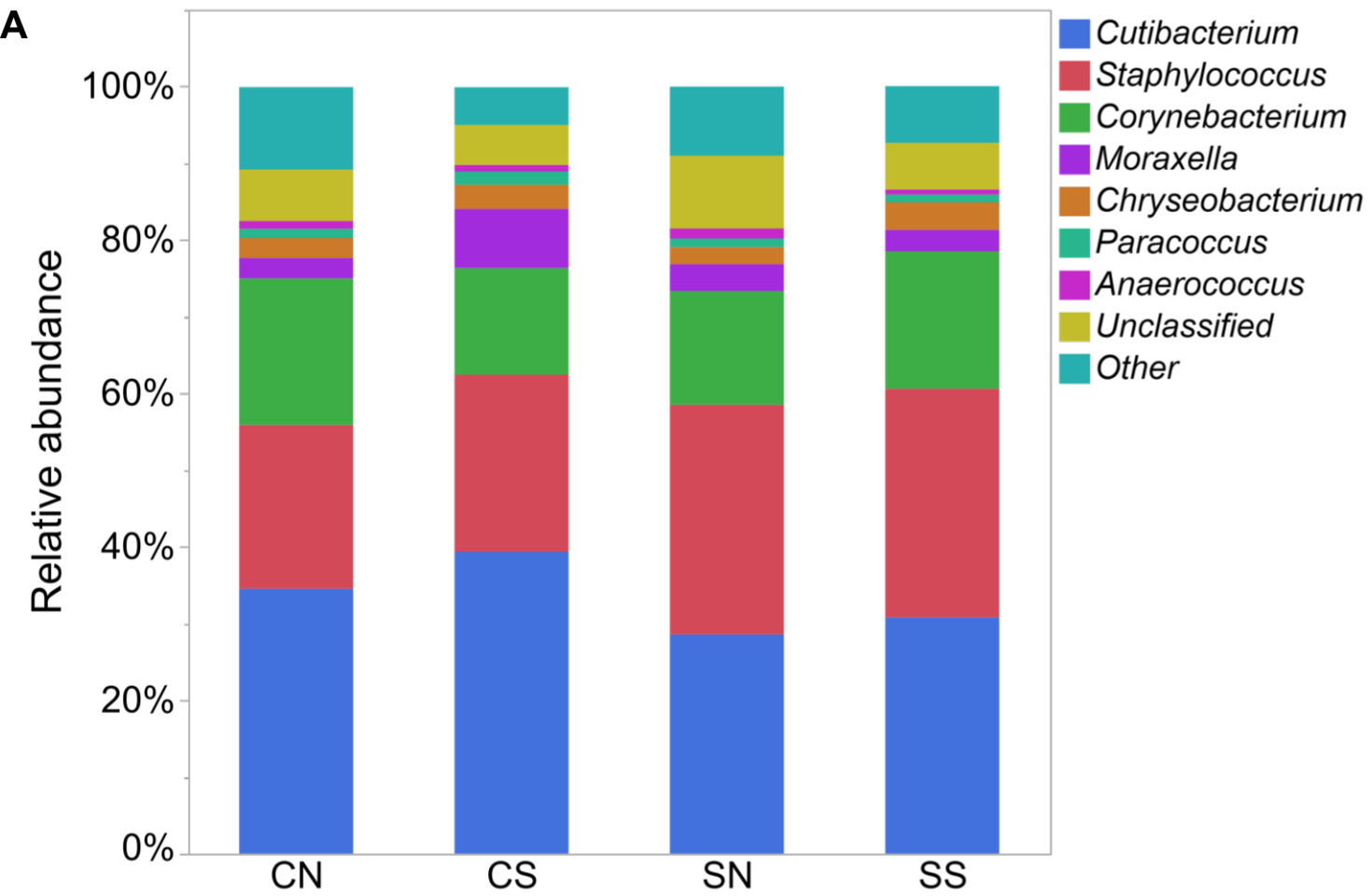**B**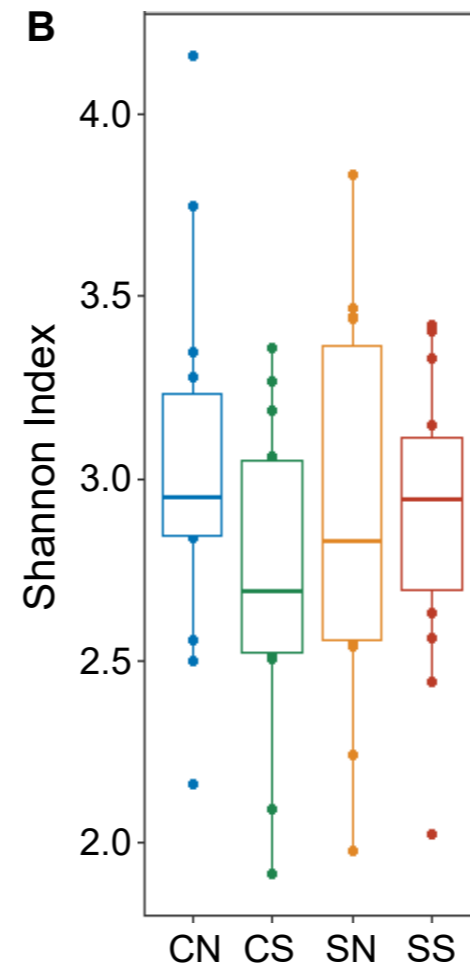**C**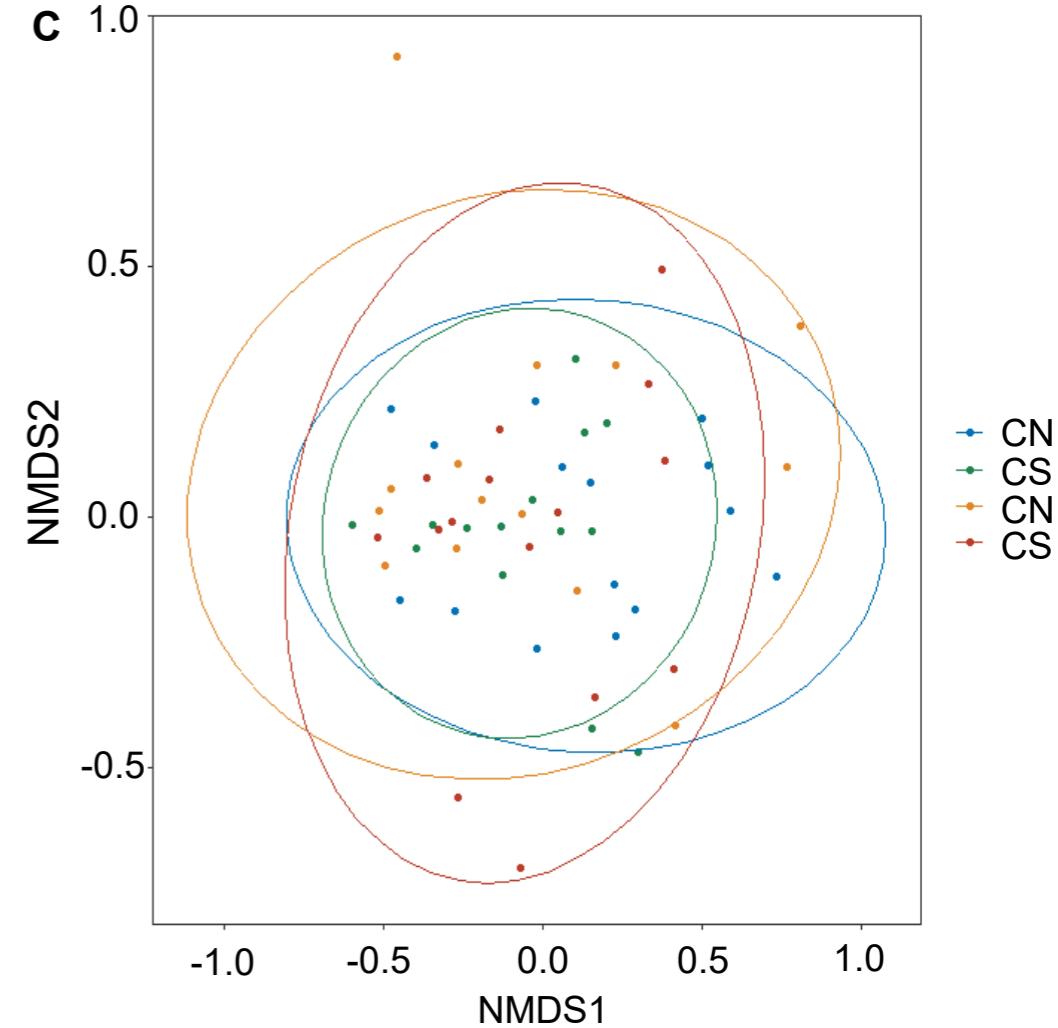

Supplement: FIG S1 [file msystems.00319-21-sf001.pdf]

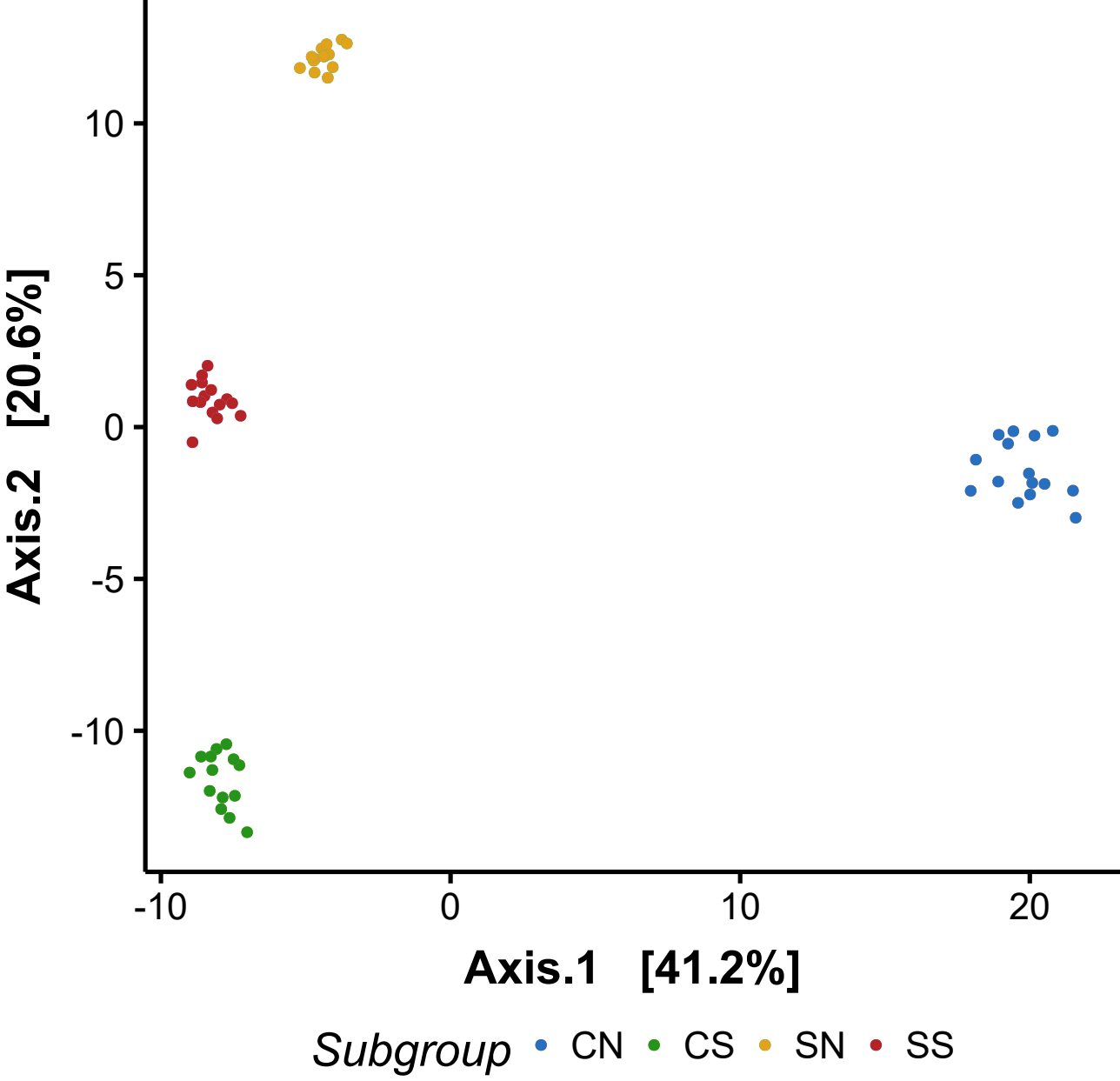

Supplement: FIG S2 [file msystems.00319-21-sf002.pdf]

**a** Dalian (low pollution)

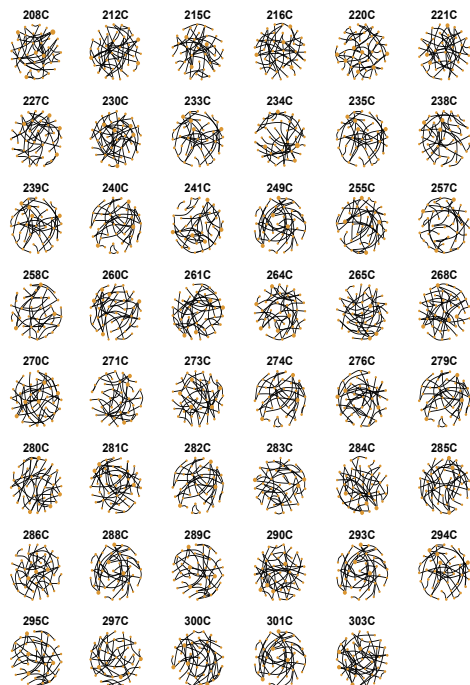

**Baoding (high pollution)**

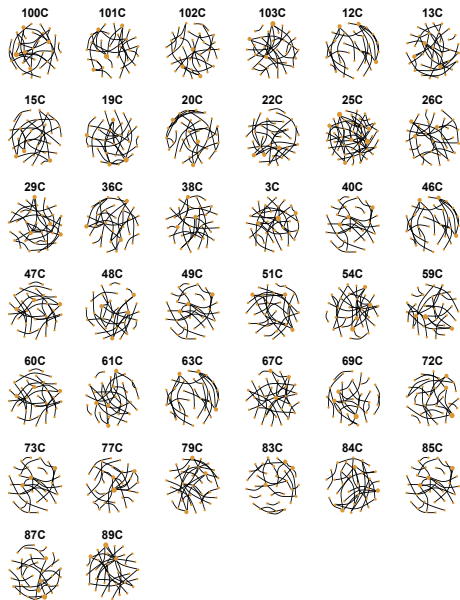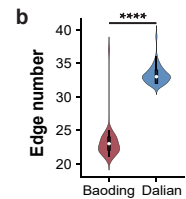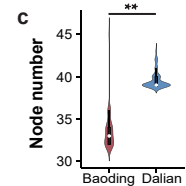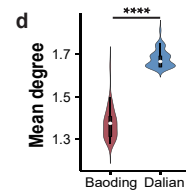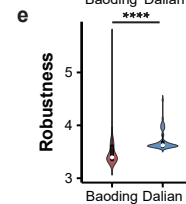

Supplement: FIG S3 [file msystems.00319-21-sf003.pdf]
