# Supplementary material for: Facial Skin Microbiota-Mediated Host Response to Pollution Stress Revealed by Microbiome Networks of Individual
Source: mSystems. 2021 Jul 27;6(4):e00319-21. doi: 10.1128/mSystems.00319-21 (PMC8407115; doi:10.1128/mSystems.00319-21)
Supplement: TABLE S1 [file msystems.00319-21-st001.docx]

| Network property | Diversity attributes | Skin indices |
| --- | --- | --- |
| Vulnerability(robustness) | observed alpha | SCTE activity |
| assortativity | chao1 | Cholesterol |
| average path | diversity inverse simpson | Decomposed H_2_O_2_ (catalase) |
| diameter | diversity gini simpson | Total antioxidant capacity |
| edgenumber | diversity shannon | Vitamin E (VE) |
| mean betweenness | diversity fisher | TEWL |
| mean degree | diversity coverage |  |
| nodenumber | evenness camargo |  |
| transitivity | evenness pielou |  |
|  | evenness simpson |  |
|  | evenness evar |  |
|  | evenness bulla |  |
