# Supplementary material for: Facial Skin Microbiota-Mediated Host Response to Pollution Stress Revealed by Microbiome Networks of Individual
Source: mSystems. 2021 Jul 27;6(4):e00319-21. doi: 10.1128/mSystems.00319-21 (PMC8407115; doi:10.1128/mSystems.00319-21)
Supplement: TABLE S3 [file msystems.00319-21-st003.pdf]

A

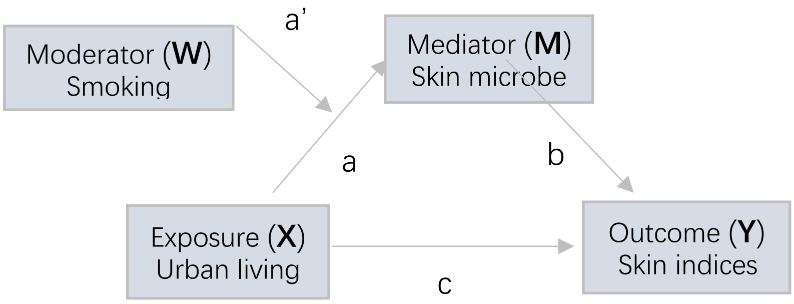

B

Cholesterol (Y)	Coeff	SE	P
Edge number (M)	0.4862	0.2013	0.0191
Location (X)	-1.5363	0.0780	0.0000
Smoking (W)	-0.9276	0.0780	0.0000
Location: Smoking (XW)	1.0662	0.1560	0.0000
R^2	0.1656		
**Cholesterol (Y)**			
Mean degree (M)	0.4260	0.1771	0.0196
Location (X)	-1.3852	0.0980	0.0000
Smoking (W)	-1.1333	0.0980	0.0000
Location: Smoking (XW)	0.6393	0.1959	0.0019
R^2	0.1649		
**Cholesterol (Y)**			
Robustness (M)	0.4813	0.2009	0.0200
Location (X)	-1.5342	0.0963	0.0000
Smoking (W)	-0.9110	0.0963	0.0000
Location: Smoking (XW)	0.7757	0.1927	0.0002
R^2	0.1643		
**Antioxidant capacity(Y)**			
Node number (M)	0.4885	0.1932	0.0144
Location (X)	-1.5008	0.0607	0.0000
Smoking (W)	-0.6117	0.0607	0.0000
Location: Smoking (XW)	2.0431	0.1214	0.0000
R^2	0.1565		

Note. *N*=58 subjects. As Hayes recommend (Bolin 2014), we code smoking/ non-smoking as 1/0, urban/ rural as 1/0.
Coeff: path coefficient,
P: The P value indicates the significance of the path.
SE: standard error.
Y: outcome
M: mediator
X: exposure
W: moderator
XW: interaction (moderation effect)

Vitamin E (Y)	Coeff	SE	P
Edge number (M)	0.5825	0.2026	0.0057
Location (X)	-1.5363	0.0780	0.0000
Smoking (W)	-0.9276	0.0780	0.0000
Location: Smoking (XW)	1.0662	0.1560	0.0000
R^2	0.1551		
**Vitamin E (Y)**			
Mean degree (M)	0.5128	0.1781	0.0057
Location (X)	-1.3852	0.0980	0.0000
Smoking (W)	-1.1333	0.0980	0.0000
Location: Smoking (XW)	0.6393	0.1959	0.0019
R^2	0.1553		
**Vitamin E (Y)**			
Robustness (M)	0.5571	0.2032	0.0082
Location (X)	-1.5342	0.0963	0.0000
Smoking (W)	-0.911	0.0963	0.0000
Location: Smoking (XW)	0.7757	0.1927	0.0002
R^2	0.1449		
**SCTE (Y)**			
Node number (M)	0.5214	0.1943	0.0096
Location (X)	-1.5008	0.0607	0.0000
Smoking (W)	-0.6117	0.0607	0.0000
Location: Smoking (XW)	2.0431	0.1214	0.0000
R^2	0.1468		
**SCTE (Y)**			
Edge number (M)	0.4721	0.2069	0.0264
Location (X)	-1.5363	0.0780	0.0000
Smoking (W)	-0.9276	0.0780	0.0000
Location: Smoking (XW)	1.0662	0.1560	0.0000
R^2	0.1185		

Supplement table 3: conditional process analysis for 4 variables

A. Illustration of conditional process model
B. Coefficient table of models that have significant moderation effect
